# Frequency of Developing COVID-19 Pneumonia in Patients Who Were Vaccinated Double-Dose CoronaVac: Data of the Pandemic Authorized Hospital in Northern Cyprus

**DOI:** 10.4314/ejhs.v33i5.2

**Published:** 2023-09

**Authors:** Ersan Berksel, Asli Aykac, Dilaver Akdur, Kaya Suer

**Affiliations:** 1 Cyprus Science University, Faculty of Health Sciences, Department of Nursing, Nicosia, Cyprus; 2 Near East University, Department of Biophysics, Nicosia, Cyprus; 3 Dr. Burhan Nalbantoglu State Hospital, Department of Radiology, Nicosia, Cyprus; 4 Near East University, Faculty of Medicine, Department of Infectious Diseases and Clinical Microbiology, Nicosia, Cyprus

**Keywords:** Chest CT, CoronaVac, COVID-19, Northern Cyprus, SARS-CoV-2

## Abstract

**Background:**

RT-PCR is the leading method used in the diagnosis of COVID-19, caused by 2019-nCoV. CT applications also provide a fast and easy diagnosis for detecting pneumonia caused by the SARS-CoV-2 virus. The current study, aimed to compare the lung involvement of vaccinated (two-dose CoronaVac) and unvaccinated patients in the early stage of COVID-19 disease.

**Methods:**

In the current retrospective study, which included patients diagnosed with RT-PCR COVID-19 positivity (n=651) between 01 July 2021-15 September 2021, patient information was obtained from the authorized hospital of the pandemic. Data included patients' chest CT scans and whether patients had been vaccinated (two-dose CoronaVac) information.

**Results:**

The ratio of vaccination with double-dose CoronaVac in positive patients was 74.3%. The ratio of patients with normal lung appearance was 61.8%. It was determined that the ratio of involvement in both lungs of patients who were vaccinated with a double dose was significantly lower than the ratio of involvement in patients who were never vaccinated (p <0.001).

**Conclusion:**

In this study, it was determined that pneumonia cases were less common in individuals vaccinated with double-dose CoronaVac. In this study, it was also determined that the protection of the vaccine was higher in females than in males and that the protection of the double-dose CoronaVac vaccine was higher in the 50-60 age group compared to 60 older patients.

## Introduction

The pandemic, which started in Wuhan City, Hubei Province, China, and spread to 33 countries in just two months, has been continuing since December 2019. In the fight against infectious diseases for which there is no specific treatment (1) or vaccine, it is essential that the infected person be immediately isolated from the healthy population in order not to pose a threat to public health. These rules were also applied during the COVID-19 pandemic, due to the unknowns about the existence of an effective treatment and/or vaccine against the virus (2, 3).

Individuals are determined to be COVID-19-positive (+) or suspected of being COVID-19-(+) after research using respiratory and/or blood samples. RT-PCR is a leading method used in the diagnosis of COVID-19 caused by 2019-nCoV. It was reported that the RT-PCR-(+) ratio was approximately 30-60% at the beginning of the pandemic (4). The low positivity ratio obtained from the PCR, the highly contagious nature of the virus, and the risk of transmission to the healthy population once again reveal the current emergency against the pandemic.

A computed tomography (CT) provides fast and easy diagnosis in the detection of pneumonia caused by the SARS-CoV-2 virus (5, 6). There are many studies reporting the relationship between the disease and lung involvement with images obtained from chest CTs of COVID-19 patients (7-10).

Following the declaration of the pandemic by the WHO, studies on developing vaccines against COVID-19 started in different countries. Countries have started to vaccinate the public, especially health workers and at-risk groups, by choosing different preferences among the vaccines produced against the pandemic. CoronaVac was the first type of vaccine used in the Northern Cyprus (NC) during this study. Individual vaccinations across the country were carried out in the following order: first to healthcare professionals, then to individuals aged 65 and older and individuals with chronic diseases. In the course of the pandemic, different vaccines such as BioNTech, Astra-Zeneca, Janssen-Johnson & Johnson, and Moderna were also applied to the public. Phase 3 studies of the CoronaVac double dose vaccine show that it is 86% effective against death, 88-100% effective against serious illness, and 65-85% effective against symptomatic disease in the general population (11). Reports from countries using CoronaVac highlight that individuals are still infected with the virus despite being vaccinated with two doses of CoronaVac (12-14).

According to the policy implemented by the authorized pandemic hospital in the NC at the beginning of the pandemic process, CT was required within 48 h for all patients 50 years of age and older who were found to have positive RT-PCR test results. In this period, according to the statements made by the Ministry of Health in the NC, the dominant variant on the island was the SARS-CoV-2-alpha (a) variant. The current study, aimed to compare the lung involvement of vaccinated (two-dose CoronaVac) and unvaccinated patients in the early stage of COVID-19 disease.

## Patients and Methods

**Study design and study population**: Ethical approval for the study was obtained from the local ethics committee of Cyprus Science University (approval no. 2021.12.001). This study was conducted as a single-center, retrospective, cross-sectional study in which patient information was obtained from the pandemic authorized hospital of the country for the diagnosis and treatment of COVID-19. The inclusion criteria were as follows: (i) RT-PCR positive detection of positive SARS-CoV-2 nucleic acid in throat/nose swabs, and (ii) at least one thin-slice CT application.

CoronaVac was one of the vaccines available in the NC at the time the research data was obtained. Priority vaccination in the country was implemented as follows: healthcare professionals, individuals over 65 years of age, and individuals with underlying diseases. In the advancing period of COVID-19, vaccination for those over 65 years of age has been reduced to 50 years of age and above. The information of individuals 50 and over 50 years of age who have the right to priority vaccination and those with underlying diseases were checked through the NC Ministry of Health information system and vaccinated at the pandemic-authorized hospital. While the information of the vaccinated persons, such as the vaccination barcode number and vaccination date, was recorded in the TRNC ministry information system, the vaccination card containing the same information was also delivered to the persons. Thus, whether the individual was vaccinated or not could be checked with the vaccination card given to them at the time of vaccination and/or ID numbers from the NC Ministry of Health information system.

**Data collection**: Between July 1 and September 15, 2021, 651 patients were diagnosed with COVID-19 by RT-PCR testing at the only authorized pandemic hospital in the country. Chest CT (Revolution EVO GE; Medical Systems, Milwaukee, WI, USA) images taken from patients with positive RT-PCR in the NC-authorized pandemic hospital were collected and evaluated by our specialist.. All patients included in this study were diagnosed with COVID-19 according to the diagnostic criteria in the COVID-19 Diagnosis and Treatment Guidelines by China (14). Data included whether patients were vaccinated with a double dose of CoronaVac and included chest CT scan images. All images were obtained with a CT device (128 slices) at the end of inspiration with the patients in the supine position. The time between the second dose vaccination of individuals and the time when COVID-19-(+) was detected was also examined. The dates on which the vaccination started according to the groups were accepted as the vaccination days of the unvaccinated patients.

**Statistical analysis**: Statistical analysis was performed on SPSS (Ver: 18.0). Descriptive statistics were given mean ± standard deviation (SD) values for continuous data, and in numbers and percentages for categorical data. A one-way ANOVA test was performed to draw conclusions from the analyzes of COVID-19 RT-PCR-(+) patients. The relationship between age, gender, region of involvement finding, and the time between the date they were found to be positive for COVID-19 and the time of vaccination was evaluated by regression analyses. p values below the 95% confidence interval and below 0.05 were considered significant.

## Results

**Description of study population**: It was determined that 327 patients were female and 324 patients were male. The youngest of the patients was 50 years old and the oldest 85 years old. The mean age was 60.44±7.9 years. When the patient records from the hospital information system were examined, it was determined that the ratio of those who were vaccinated with two doses of CoronaVac was 484. The time elapsed between the date of detection of COVID-19-(+) and the time of vaccination (time since vaccination) was given in Table 1. Accordingly, 37.3% of the individuals were found to be COVID-19-(+) 12-18 months after vaccination (Table 1).

**Table 1:** Information on the demographic features of the PCR-positive patients included in the study, whether they were vaccinated double-dose CoronaVac or not and time since vaccination

Parameters (n=651)		Number (n)	Percentage (%)
Gender	Female	327	50.3
	Male	324	49.7
Age groups (years)	50-60	378	58.1
	61-70	182	28.0
	70^+^	91	13.9
Double dose CoronaVac	-	167	25.7
	+	484	74.3
Time since vaccination (month)	1-3	35	5.4
	4-6	75	11.5
	7-9	112	17.2
	10-12	186	28.6
	13-18	243	37.3
Total		651	100

**Chest CT images analysis**: CT was taken to evaluate the general condition of the patient, aged 50 years and older, whose RT-PCR test was positive, and to evaluate early lung involvement, in line with the sanctions of the NC Ministry of Health. Lung involvements according to chest CT results in vaccinated or unvaccinated patients were given in Table 2.

**Table 2:** Lung involvements according to chest CT results in vaccinated or unvaccinated patients

COVID-19 RT-PCR test positive patients (n=651)		double-dose CoronaVac vaccine (n=484)		no vaccine (n=167)		P
		**n**	**%**	**n**	**%**	
Involvement	Yes	167	34.5	81	48.5	
	No	317	65.5	86	51.5	0.01**
Findings	Normal	317	65.5	86	51.5	
	Unilateral	70	14.5	23	13.8	
	Bilateral	97	20.0	58	34.7	0.05*
Affected lung lobe	None	317	65.5	86	51.5	
	1	56	11.6	18	10.8	
	1^+^	111	22.9	63	37.7	0.05*

* p < 0.05

** p < 0.001 vs vaccinated and unvaccinated positive patients

**Figure 1:**
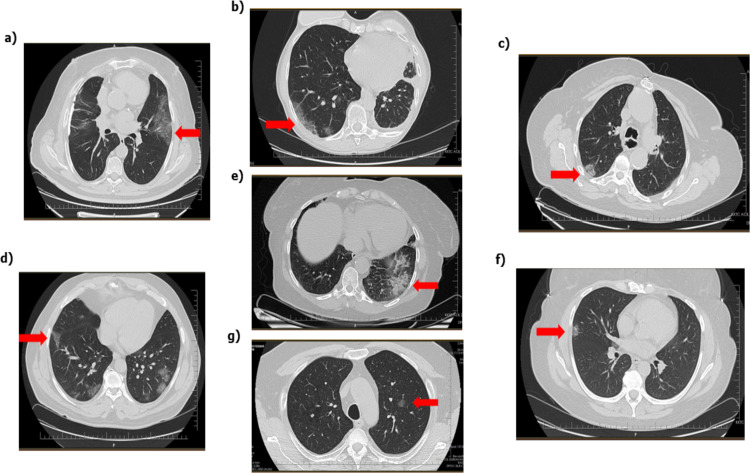
Various lesions of COVID-19 RT-PCR positive patients whose CT images were retrospectively analyzed. a) Ground-glass images of the left lung with lingula septal thickening and air bronchograms b) Ground-glass images of the right lung with the sub-pleural location at the base and involvements accompanied by septal thickenings. c) Ground-glass images of peripheral subpleural and peribronchovascular in the upper lobe of the right lung. d) Central focal ground-glass view in the apicoposterior segment of the left lung upper lobe. e) Cobblestone images in the left lung basal. f) Ground-glass densities accompanied by septal thickenings with widespread involvement in the lower lobe and right middle lobe of both lungs. g) Consolidation area with peripheral involvement in the right lung middle lateral segment

While 34.5% of the vaccinated patients had lung involvement, 65.5% did not have any involvement in their lungs. It was found that the ratio of involvement in the lungs of vaccinated patients was significantly lower than the ratio of involvement in the lungs of unvaccinated patients (p < 0.01). Unilateral lung involvement was found in 13.8% of the vaccinated patients, and bilateral lung involvement in 34.7%. Bilateral involvement ratios in vaccinated patients were considerably lower than in unvaccinated patients (p < 0.05). While 11.6% of the vaccinated patients were involved in one lobe of the lung, involvement of more than one lung lobe was observed in 22.9%. (Table 2 and Figure 1). About 10.8% of unvaccinated patients were involved in one lobe of the lung, while the involvement of more than one lobe of the lung was observed in 37.7% of them. 14.5% of the vaccinated patients had unilateral lung involvement, while bilateral lung involvement was determined in 20.0% of them. It was determined that the ratio of involvement of more than one lobe in the lungs of vaccinated patients was significantly lower than the rates of unvaccinated patients (p < 0.05).

It was determined that the ratio of lung involvement was almost the same in unvaccinated female and male patients (50.0% and 47.1%, respectively; Table 3). Similarly, the ratio of detecting any involvement in the lungs of vaccinated female and male patients was determined to be almost the same in both genders (52.9% and 50.0%, respectively). In vaccinated female patients, the ratio of lung involvement was 29.8%. This ratio was 39.3% for vaccinated men (Table 3).

**Table 3:** CT lung involvement in COVID-19 RT-PCR test-positive patients by gender

		Two doses CoronaVac vaccine			

		+		-	
				
Gender		female n (%)	male n (%)	female n (%)	male n (%)
Involvement	+	73 (29.8)	94 (39.3)	41 (50.0)	40 (47.1)
	-	172(70.2)^+^	145 (60.7)	41 (50.0)	45 (52.9)
Findings	unilateral	30 (12.2)	40 (16.7)	16 (19.5)	7 (8.2)
	bilateral	43 (17.6)**	54 (22.6)*	25 (30.5)	33 (38.8)

* p < 0.05

** p < 0.001 vs vaccinated and unvaccinated positive patients, ^+^p < 0.05 vs gender

It was found that the ratio of any involvement in vaccinated female patients was significantly lower than the ratio of involvement in vaccinated male patients (p < 0.05). The bilateral lung involvement ratio in vaccinated males was 22.6%, while the ratio of bilateral lung involvement in unvaccinated males was 38.8%. The ratio of bilateral lung involvement in vaccinated females was 17.6% and 30.5% in unvaccinated females. It was determined that the bilateral involvement ratio in female and male vaccinated patients was significantly lower than the bilateral involvement ratio in unvaccinated female and male patients (p < 0.01 for females and p < 0.05 for males).

The ratio of lung involvement in vaccinated and unvaccinated patients aged 50–60 years was 27.9% and 53.3%, respectively (Table 4). Significantly less lung involvement was detected in double-dose CoronaVacvaccinated patients in the 50–60 age group compared to the unvaccinated patients (p < 0.01). In vaccinated patients aged 50–60 years, the bilateral lung involvement ratio was 14.3%, while it was 39.2% in unvaccinated patients. The ratio of bilateral involvement detected in vaccinated patients in the 50–60 age group was found to be significantly less than that in the unvaccinated patients (p <0.05). In vaccinated patients aged 61–70 years, the bilateral lung involvement ratio detected was 26.9%, while it was 20.0% in unvaccinated patients. In vaccinated patients aged 71 and older, the bilateral involvement ratio detected was 25.7%, while it was 29.4% in unvaccinated patients (Table 4). There was no significant difference between the age groups and the rates of bilateral lung involvement in patients who were vaccinated or unvaccinated.

**Table 4:** CT lung involvement in COVID-19 RT-PCR test-positive patients by age groups

		Age groups (years)					

		50-60 (n=378)		61-70 (n=182)		70^+^ (n=91)	
Two doses CoronaVac vaccine		+	-	+	-	+	-
		n (%)	n (%)	n (%)	n (%)	n (%)	n (%)
Findings Involvement	+	72 (27.9)	64 (53.3)	62 (40.8)	10 (33.3)	33 (44.6)	7 (41.2)
	-	186 (72.1)**	56 (46.7)	90 (59.2)	20 (66.7)	41 (55.4)	10 (58.8)
	unilateral	35 (13.6)	17 (14.2)	21 (13.8)	4 (13.3)	14 (18.9)	2 (11.7)
	bilateral	37 (14.3)*	47 (39.2)	41 (26.9)	6 (20.0)	19 (25.7)	5 (29.4)

* p < 0.05

** p < 0.01 vs vaccinated and unvaccinated positive patients

When the model based on the relationship between age, gender, site of involvement and the time elapsed since vaccination was evaluated by regression analysis, the following results were obtained: the power of the model (R^2^) was 0.787 and the significance was determined as p < 0.001. A positive correlation was found between the time since vaccination, gender (β= 0.103) and lung involvement (β= 0.652) and a negative correlation with age (β= -0.198) (Table 5). It was determined that the relationship between the time since vaccination and gender, the time of detection of COVID-19 positive in female patients was significantly later than in male patients (p < 0.01). When evaluating the relationship between the time since vaccination and age group, it was determined that the duration of being positive for COVID-19 in elderly patients was significantly earlier than in patients aged 50-60 years (p < 0.001). When the relationship between the time since vaccination and involvement finding was evaluated, it was determined that the bilateral involvement rate in patients with double-dose CoronaVac vaccine was significantly lower than in non-vaccinated patients (p < 0.001).

**Table 5:** Results from the regression model

Independent Variables	β	t	P
Time since vaccination (Constant)	4.251	14.450	0.001
Gender	0.103	3.539	0.01**
Age	-0.198	-6.987	0.001***
Involvement findings	0.652	23.081	0.001***

** p < 0.01 vs time since vaccination and gender, age and involvement findings

*** p < 0.001 vs time since vaccination age and involvement findings

## Discussion

After the declaration of the pandemic, different types of vaccine studies were started against COVID-19 infection. CoronaVac was the first type of vaccine used in the NC during this study. Individual vaccinations across the country were carried out in the following order: first to healthcare professionals, then to individuals 65 and older, and individuals with chronic diseases. Phase 3 studies of double-doseCoronaVac show that it is 86% effective against death, 88-100% effective against serious illness, and 65-85% effective against symptomatic disease in the general population (11). Reports from countries using CoronaVac highlight that individuals were still infected with the virus despite being vaccinated with two doses of CoronaVac (12, 13, 15).

In a study conducted in India between May and July 2021, the ratio of COVID-19 infection among individuals vaccinated with double-dose COVAXIN was 10% (16). In the current study, it was aimed to evaluate whether 651 patients with a positive COVID-19 RT-PCR test were vaccinated with double-dose CoronaVac and to evaluate the efficacy/protection of the double-dose CoronaVac against the virus with images obtained from CT. In our study results, it was determined that 51.5% of unvaccinated patients and 65.5% of vaccinated patients did not have any lung involvement. It may be possible to say that double-dose CoronaVac reduced lung involvement. In addition, it was determined that the ratio of involvement in the bilateral lobes of the lung in vaccinated patients was significantly lower than the bilateral involvement ratio in unvaccinated patients. According to our findings, it may be possible to say that two doses of the CoronaVac prevent or protect against the involvement of the virus in the lungs. In our findings, it was determined that the ratio of involvement in the bilateral lobes of the lung in vaccinated patients was significantly lower than in unvaccinated patients. Based on this finding, it seems possible to suggest that the double-dose CoronaVac prevent double involvement in the lung. In our study, it was determined that the ratio of patients not having any involvement in the lungs of vaccinated female patients was higher than that of vaccinated males. The double dose of CoronaVac may have provided higher protection in females than in males. In the results of the study with CoronaVac, it is emphasized that there is a decrease in the antibody titer formed in the body in correlation with the increase in age (17). In our study, it was determined that the bilateral involvement ratio of the vaccinated patients in the 50–60 age group was lower compared to the patients in other age groups. It can be interpreted as an indication that vaccination after the age of 60 is ineffective.

Although the RT-PCR method is considered the gold standard in the diagnosis of COVID-19, this method has a 10-40% false-negative probability. Therefore, chest CT is recommended for both diagnosis and treatment planning in addition to RT-PCR tests for individuals admitted to hospitals due to COVID-19 (18-21).

It was reported that 56.4% of the 3062 patients evaluated were male in a meta-analysis study (22). In our study result, it was determined that the number of COVID-19-(+) female (50.3%) patients was higher than the number of male (49.7%) patients, but this difference was not significant. Sharif et al., stated that bilateral lung involvement was detected in 5220 (78%) of the cases evaluated in the systematic review study conducted (23). In the same article, the ratio of involvement in more than one lung lobe was reported as 75%. A meta-analysis study stated that 25.8% of the patients had unilateral lung involvement and 75.7% had bilateral lung involvement (22). In a study evaluating 83 unvaccinated pneumonia cases, the rate of bilateral involvement was found to be 95.2% (24). Radiological abnormalities increase over the disease course, and the typical peak is at 10-12 days post-symptom initiation. At early stages or in mild disease, imaging may not reveal any pathology, but interestingly abnormal findings on imaging can be identified in some cases prior to symptom development or even prior to PCR RNA detection (25).

In our results, bilateral involvement was detected in only 23.8% and unilateral involvement in 14.3% of COVID-19-(+) patients. Bilateral involvement ratio was found to be lower in vaccinated patients (20.0%) compared to unvaccinated (34.7%) patients. The fact that 74.3% of vaccinated patients suggests that it may be effective in the low ratio of bilateral involvement seen in CT. These data support the argument that bilateral involvement is less in COVID-19-(+) cases with vaccinated pneumonia. Although it is obvious that the double-dose CoronaVac cannot completely prevent COVID-19 infection, it is possible to say that double-dose CoronaVac vaccinated individuals have a milder course of the disease and a lower incidence of pneumonia.

In our study with vaccinated patients; only one of the left or right lung lobes were affected in 75 patients, one and more lobes in 174 patients. In a study conducted at a time when vaccination had not yet started, when the rates of involvement in the lung lobes of patients who developed pneumonia were examined, 19 (30.2%) one lung lobe was affected, two lung lobes were affected 5 (7.9%), three or more lung lobes were affected 39 (61.8%) people were reported (19). In the same study, while the number of lobes affected by the virus in COVID-19-(+) individuals was determined as 3.3±1.8, this ratio was determined as 1.37±0.8 in vaccinated patients in our study. It may be possible to say that the involvement of the lung lobes in the case of COVID-19-(+) in vaccinated people was less than in unvaccinated patients.

The efficacy ratio of CoronaVac is reported with different ratios in various countries in the literature. It was determined that lung involvement in the early stages of COVID-19 was less common in individuals vaccinated with double-dose CoronaVac. It was also determined that the protective effect of the vaccine on the lungs was higher in females than in males.

Our study results include the first findings of NC, as well as the addition of a new one to the reports reporting the effects of double-dose CoronaVac vaccine on pneumonia cases with different results. The limitation of our research is, there remains a need for new studies that both include COVID-19 patients after September 15, 2021 and determine the effectiveness of other vaccines arriving on the island later.

## References

[1] Maharajan MK, Rajiah K, Pogula B, Chigurupati S, Katragadda S (2022). Pharmaceutical, biological agents, and vaccines under clinical trials for COVID-19 and roles of pharmacists to combat COVID-19, an update. J Res Pharm.

[2] WHO Director-General's remarks at the media briefing on 2019-nCoV on 11 February 2020.

[3] Zhu N, Zhang D, Wang W, Li X, Yang B, Song J, China Novel Coronavirus Investigating and Research Team (2020). A Novel Coronavirus from Patients with Pneumonia in China, 2019. N Engl J Med.

[4] Long C, Xu H, Shen Q, Zhang X, Fan B, Wang C (2020). Diagnosis of the Coronavirus disease (COVID-19): rRT-PCR or CT?. Eur J Radiol.

[5] Lv M, Wang M, Yang N, Luo X, Li W, Chen X (2020). Chest computed tomography for the diagnosis of patients with coronavirus disease 2019 (COVID-19): a rapid review and meta-analysis. Ann Transl Med.

[6] Inui S, Gonoi W, Kurokawa R, Nakai Y, Watanabe Y, Sakurai K (2021). The role of chest imaging in the diagnosis, management, and monitoring of coronavirus disease 2019 (COVID-19). Insights Imaging.

[7] Caruso D, Zerunian M, Polici M, Pucciarelli F, Polidori T, Rucci C (2020). Chest CT Features of COVID-19 in Rome, Italy. Radiology.

[8] Pan F, Ye T, Sun P, Gui S, Liang B, Li L (2020). Time Course of Lung Changes at Chest CT during Recovery from Coronavirus Disease 2019 (COVID-19). Radiology.

[9] Poortahmasebi V, Zandi M, Soltani S, Jazayeri SM (2020). Clinical Performance Of RT-PCR and Chest CT Scan for Covid-19 Diagnosis; A Systematic Review. Adv J Emerg Med.

[10] Ocak M, Yurt NŞ, Yurt YC (2021). Examination of clinical conditions and chest CT images of Covid-19 cases in Turkey; single center study. Afr Health Sci.

[11] Higdon MM, Wahl B, Jones CB, Rosen JG, Truelove SA, Baidya A (2022). A Systematic Review of Coronavirus Disease 2019 Vaccine Efficacy and Effectiveness Against Severe Acute Respiratory Syndrome Coronavirus 2 Infection and Disease. Open Forum Infect Dis.

[12] Tanriover MD, Doğanay HL, Akova M, Güner HR, Azap A, Akhan S (2021). CoronaVac Study Group. Efficacy and safety of an inactivated whole-virion SARS-CoV-2 vaccine (CoronaVac): interim results of a double-blind, randomised, placebo-controlled, phase 3 trial in Turkey. Lancet.

[13] Palacios R, Patiño EG, de Oliveira Piorelli R, Conde MTRP, Batista AP, Zeng G (2020). Double-Blind, Randomized, Placebo-Controlled Phase III Clinical Trial to Evaluate the Efficacy and Safety of treating Healthcare Professionals with the Adsorbed COVID-19 (Inactivated) Vaccine Manufactured by Sinovac - PROFISCOV: A structured summary of a study protocol for a randomised controlled trial. Trials.

[14] Çoban MM, Yakar MN, Küçük M, Ergün B, Çelik M, Ergan B (2022). COVID-19 Pneumonia After SARS-CoV-2 Vaccination with CoronaVac: A Case Series from Turkey. Turk Thorac J.

[15] National Health Commission of the PRC (2020). The Guidelines for the Diagnosis and Treatment of New Coronavirus Pneumonia.

[16] Singh CM, Singh PK, Naik BN, Pandey S, Nirala SK, Singh PK (2022). Clinico-Epidemiological Profile of Breakthrough COVID-19 Infection among Vaccinated Beneficiaries from a COVID-19 Vaccination Centre in Bihar, India. Ethiop J Health Sci.

[17] Fernández J, Bruneau N, Fasce R, Martín HS, Balanda M, Bustos P (2022). Neutralization of alpha, gamma, and D614G SARS-CoV-2 variants by CoronaVac vaccine-induced antibodies. J Med Virol.

[18] Tan W, Zhao X, Ma X, Wang W, Niu P, Xu W (2020). A Novel Coronavirus Genome Identified in a Cluster of Pneumonia Cases - Wuhan, China 2019-2020. China CDC Wkly.

[19] Pan Y, Guan H, Zhou S, Wang Y, Li Q, Zhu T (2020). L. Initial CT findings and temporal changes in patients with the novel coronavirus pneumonia (2019-nCoV): a study of 63 patients in Wuhan, China. Eur Radiol.

[20] Chung M, Bernheim A, Mei X, Zhang N, Huang M, Zeng X (2020). CT Imaging Features of 2019 Novel Coronavirus (2019-nCoV). Radiology.

[21] Pan F, Ye T, Sun P, Gui S, Liang B, Li L (2020). Time Course of Lung Changes at Chest CT during Recovery from Coronavirus Disease 2019 (COVID-19). Radiology.

[22] Zhu J, Ji P, Pang J, Zhong Z, Li H, He C (2020). Clinical characteristics of 3062 COVID-19 patients: A meta-analysis. J Med Virol.

[23] Sharif PM, Nematizadeh M, Saghazadeh M, Saghazadeh A, Rezaei N (2022). Computed tomography scan in COVID-19: a systematic review and meta-analysis. Pol J Radiol.

[24] Li K, Wu J, Wu F, Guo D, Chen L, Fang Z (2020). The Clinical and Chest CT Features Associated With Severe and Critical COVID-19 Pneumonia. Invest Radiol.

[25] Gavriatopoulou M, Korompoki E, Fotiou D, Ntanasis-Stathopoulos I, Psaltopoulou T, Kastritis E (2020). Organ-specific manifestations of COVID-19 infection. Clin Exp Med.

